# Examining Masculinities to Inform Gender-Transformative Violence Prevention Programs: Qualitative Findings From Rakai, Uganda

**DOI:** 10.9745/GHSP-D-21-00137

**Published:** 2022-02-28

**Authors:** Eunhee Park, Samuel Jason Wolfe, Fred Nalugoda, Lindsay Stark, Neema Nakyanjo, William Ddaaki, Charles Ssekyewa, Jennifer A. Wagman

**Affiliations:** a Fielding School of Public Health, University of California Los Angeles, Los Angeles, CA, USA.; b Mailman School of Public Health, Columbia University, New York, NY, USA.; c Rakai Health Sciences Program, Kalisizo, Uganda.; d Brown School, Washington University, St. Louis, MO, USA.

## Abstract

**Introduction:**

Evidence-based programs are needed to engage men and boys that encourage the transformation of concepts of masculinity that uphold patterns of intimate partner violence (IPV). This study explores the constructs of masculinity and male gender norms surrounding sexual attitudes and IPV among men and boys living in Rakai, Uganda.

**Methods:**

Between April and August 2017, we conducted 38 interviews and 5 focus group discussions with men and boys aged 15 to 49 years and 4 focus groups with key stakeholders to understand how male use of violence is influenced by personal, community, and society-level concepts of masculinity. We adapted 2 constructs of masculinities, reputation and respectability, in the analysis to examine masculinities in relation to IPV in the rural Ugandan setting.

**Results:**

Findings suggest men and boys upheld 2 types of masculinities: respectability versus reputation. Masculine attributes related to respectability (referred to as “responsible men”) included having a job, house, wife, and many children and taking care of family. Masculine attributes related to reputation (referred to as “cool men”) included having sexual prowess, multiple sexual partners, and the resources to buy nice things. Both masculine norms were used to justify dominance over women and IPV. The divergence of masculinity was observed among youth and young adults who participated in a gender-transformative program, suggesting the effectiveness of the program.

**Discussion:**

The gender-transformative approach should provide men and boys a chance to reflect on dynamic, often conflicting, images of a man and should empower individuals to renegotiate and reconceptualize masculine norms. This critical reflection on masculinity, which resonates with men and boys, needs to be included when engaging men within HIV and IPV prevention programs.

## Abstract (precis)

While the majority of men in rural Uganda upheld 2 conflicting masculine norms that are conceptualized as reputation (“cool man”) and respectability (“responsible man”), men in younger age groups who participated in a gender-transformative program expressed gender-equitable beliefs and attitudes.

## Teaser

**Key Findings:**

- Two conflicting masculine norms of reputation and respectability coexisted in the lives of boys and men depending on the reference group (male peers vs. community and family). Both types of masculinities endorsed violence against women by placing masculinity in a position of power.
- Reputational masculine norms (“cool masculinity”) included becoming sexually active by age 18, being attracted to women, and having high sexual stamina while respectable masculinity (“responsible masculinity”) was marked by a man’s ability to find a female partner, get married, and have children.
- Boys and young men who participated in a gender-transformative program expressed gender-equitable beliefs and attitudes, suggesting the effectiveness of the gender-transformative programming.

**Key Implications:**

- Public health educators in gender-transformative violence prevention programs need to address the male perception of dynamic, often conflicting, images of men and empower individuals to renegotiate and reconceptualize the masculine norms.
- Researchers and violence prevention program managers in community-based HIV service programs should consider incorporating a comprehensive gender-transformative program designed for boys and men. The program content could include condom use, faithfulness, male circumcision, HIV testing, enrolling, or staying in care if HIV positive, treating partners with respect, and caring about health and well-being.
- Policy makers should consider integrating gender-transformative interventions that challenge existing masculine norms and incorporate multilevel approaches that target personal, interpersonal, communal, and societal levels of change.

## INTRODUCTION

Male-perpetrated violence against women (VAW) and girls, particularly intimate partner violence (IPV), is associated with gender norms that promote male dominance and control.^1^^,^^2^ Global estimates suggest 30% of all women aged 15 years and older experience IPV at least once in their lifetime and 31% of men report lifetime perpetration.^3^^,^^4^ IPV is the leading cause of homicide in women and is associated with adverse reproductive health outcomes, as well as increased risk for injury, emotional trauma, depression, and suicide.^3^^,^^5^^,^^6^ According to the Global Health Observatory, the African region has the world’s highest lifetime prevalence of IPV among ever-partnered women (33%; 95% confidence interval [CI]=29%, 37%).^7^ In Uganda, lifetime and past-year prevalence of physical and/or sexual IPV against women is 45% and 26%, respectively.^7^

Given the high prevalence of IPV against women and girls, which represents a significant violation of women’s and girls’ rights and a public health burden, the development of effective prevention programming aimed at reducing the perpetration of IPV is a global public health priority. A range of different approaches targeting men and boys in violence prevention activities have been designed and implemented over the past 15 years, including gender-transformative programming.^8^ A 2007 World Health Organization (WHO) report examining interventions with men to address HIV, gender-based violence, and sexual and reproductive health showed that gender-transformative approaches were more effective than gender-sensitive or gender-neutral approaches.^9^ WHO and partners affirm their commitment to “gender-transformative and inclusive” approaches to eliminate VAW by 2030.^10^ Gender-transformative interventions seek to transform gender constructs and norms to improve health outcomes, reduce IPV, and improve gender equity. A recent systematic review found that gender-transformative interventions that foster gender equality and change in dominant norms of masculinity can be successful in preventing IPV, modifying inequitable attitudes toward women and power, and increasing protective sexual behaviors.^11^

A 2007 WHO report examining interventions with men showed that gender-transformative approaches were more effective than gender-sensitive or gender-neutral approaches.

Six interventions in Africa have shown positive outcomes related to social norms change and violence: Stepping Stones, Men as Partners, Phaphama Initiative in South Africa, Male Norms Initiative in Ethiopia, PATH in Kenya, and SASA! in Uganda.^12^^–^^16^ The inclusion of men and boys in gender-transformative programming acknowledges the gendered power differentials present in most societies. These programs accommodate the fact that shifts in gender norms on interpersonal, community, and societal levels will require changes in attitudes and behaviors from both men and women. A small number of such interventions have undergone rigorous evaluation of their effectiveness, such as a randomized controlled trial (RCT).^16^^–^^18^ Thus, the known impact of this type of programming on reducing IPV in Africa is limited, but available evidence suggests such approaches have promise (Box).

BOX **Randomized-Control Trials of Gender-Transformative Interventions in 3 African Countries**

Stepping Stones (2003–2006),^16^ an HIV-prevention education program to improve sexual health and gender-equitable relationships in rural South Africa found:

- No reduction in incidents of HIV (adjusted incidence rate ratio [aIRR]=0.95; 95% CI=0.67 1.38)
- No reduction in women’s self-reported intimate partner violence (IPV) victimization (adjusted odds ratio [aOR]=1.14; 95% CI=0.77, 1.68)
- Reduction in incidents of herpes simplex virus-2 infection (aIRR=0.67; 95% CI=0.47-0.97)
- Reduction in men’s self-reported IPV perpetration (aOR=0.62; 95% CI=0.38, 1.01).

There are limitations in validity of self-reported data for behaviors that are not socially desirable, and it is common to observe discrepancies between male and female reporting on IPV. When there is a discrepancy between male and female reporting, female reporting is likely to be more valid than male reporting.

Unite for a Better Life (2014–2018),^17^ a group-based participatory intervention in Ethiopia delivered to men, women, and couples found:

- Decreased reports of IPV perpetration among men when the intervention was delivered to men separately (male perpetration of past year sexual IPV: aOR=0.73; 95% CI=0.56, 0.94)
- No decrease when delivered to couples or women

Engaging Men through Accountable Practice (2016–2018),^18^ a group-based discussion series in Democratic Republic of the Congo targeting both men and women found:

- Reduction in men’s intention to commit violence (odds ratio [OR]=0.59; 95% CI=0.51, 0.67)
- No reduction in women participants’ experience of IPV (OR=0.95; 95% CI=0.81, 1.09)

In Rakai, Uganda, the Safe Homes and Respect for Everyone (SHARE) Project integrated an IPV prevention intervention with an existing HIV care, treatment, and prevention intervention.^19^ The SHARE Project Trial (2005–2009), different from the 3 RCTs previously mentioned, found significant associations between exposure to the SHARE intervention and decreased self-reported victimization of physical and sexual IPV (including forced sex) among women but no significant impact on men’s self-reports of IPV perpetration.^20^

Over the past 25 years, the Rakai Health Sciences Program (RHSP) has generated a substantial body of literature to understand the epidemiology of male-perpetrated IPV against Ugandan women and to develop effective prevention programming, including the SHARE trial. SHARE was nested into RHSP’s existing HIV research and service provision infrastructure and used the ongoing Rakai Community Cohort Study (RCCS) for data collection. Although SHARE was successful at reducing women’s reported physical and sexual experiences of IPV, the effect on men’s self-reported physical and sexual perpetration was null.^20^ It is possible that the null effect among men can be partially explained by men’s underreporting of violent behavior due to social desirability bias at both baseline and follow-up and stigma associated with disclosing abuse^20^ or lower doses of the intervention because men were less likely to engage in SHARE’s community-based activities than women.^21^ Reasons for male disengagement in SHARE were concerns about time commitment, competing work and domestic requirements, and stigma surrounding participation in the program developed for women’s rights.^22^

Despite an increased focus on gender-transformative programming and increasing evidence that suggests this type of intervention can reduce IPV, we have yet to fully discern which aspects of this type of intervention are effective at reducing IPV perpetration. Qualitative research conducted on existing dominant norms of masculinity in Uganda and its relation to HIV showed having higher social status, economic well-being, power, and promiscuity supports commoditization of women and disparages messages of HIV prevention efforts.^23^ Siu et al. used 2 concepts of masculinity to understand barriers and facilitators to HIV testing in rural Uganda and found men subscribed to the traditional role of father and provider were motivated to seek HIV care and treatment.^24^^–^^26^

Despite increasing evidence that suggests gender-transformative interventions can reduce IPV, we have yet to fully discern which aspects of these interventions are effective at reducing IPV perpetration.

This study seeks to expand on lessons learned during the implementation and evaluation of SHARE^19^^,^^22^ by responding to current gaps in knowledge of men’s use of violence in relation to masculinity in Rakai, such as how men and boys perceive masculinities and what aspects of masculinities are related to violence perpetration and justification. We aim to provide a contextualized understanding of the social constructs of masculinity surrounding violence perpetration in the rural Rakai region of Uganda.

## METHODS

### Data Collection

All participants (N=111) (Table) were recruited via RCCS, an open cohort, longitudinal HIV surveillance study that RHSP established in 1994. Details about the RCCS have been published previously.^20^^,^^27^^,^^28^ The RCCS consists of a demographic census, the recording of household GPS coordinates, a quantitative survey questionnaire to assess demographics, sexual and health-seeking behaviors and HIV service uptake, and biological testing of HIV and other sexually transmitted infections. All RCCS participants are offered free HIV testing and post-test counseling. The RCCS enrolls individuals aged 15–49 years in 40 community clusters in the Rakai region.^27^^–^^29^ Eligible RCCS participants were asked for permission to be recontacted by RHSP staff members for recruitment in future research studies, including the current study.

**TABLE.** Participants in Qualitative Study on Examining Masculinity and Male Gender Norms Related to Intimate Partner Violence in Rakai, Uganda (N=111)

	**No. (%)**
In-depth interviews (n=38)	
Age range of male community members, years	
15–17	8 (21.1)
18–24	11 (28.9)
25–49	10 (26.3)
Age range of male local leader	
25–49	9 (23.7)
Focus group discussions (9 focus groups; n=73)	
Age range of male community member, years	
15–17: 2 boys groups	21 (28.8)
18–24: 2 young men groups	20 (27.4)
25–49: 1 adult men group	8 (11.0)
Key informant	
RHSP counselors and health educators: 2 mixed gender groups	11 (15.1)
Male local leaders: 2 male-only groups^a^	13 (17.8)

a The age of local leader FGD participants ranged from 25 to 67.

Researchers recruited current study participants from the RHSP Social and Behavioral Studies Department using a purposive sampling approach to enroll male community members and key informants. We used qualitative in-depth interviews (IDIs) and focus group discussions (FGDs) for data collection and analysis. Criteria for inclusion in the male community member IDIs included being male, aged 15–49 years, and a resident of an RCCS community. Male community member FGD participants were stratified by age into 3 categories: boys (aged 15–17 years), young men (aged 18–24 years), and adult men (aged 25–49 years). Criteria for inclusion in the key informants FGDs included being an active local leader, RHSP counselor, or health educator. Age was not considered for the eligibility criteria for key informants in the FGDs. Acquaintances between FGD participants were not assessed during the recruitment or discussion process. FGD participants were asked to keep confidentiality of the discussion and made aware of the limitations of confidentiality in the group discussion when providing informed consent because the study was conducted in a rural setting where participants may know one another. All study participants were from 4 agrarian communities and 1 trading community in Rakai. All IDIs and FGDs were conducted using a semistructured interview guide to allow for probing and in-depth discussion of topics of interest.

Between April and August 2017, we first conducted IDIs with community members (male; n=38) to acquire individual-level information about participants’ perceptions on masculinity, male gender norms surrounding sexual attitudes and behaviors, and the association of these norms and attitudes with IPV.

Then, informed by topics and themes identified from the IDIs, we conducted FGDs with individuals (n=73) to expand on findings from IDIs by gathering community-level information on masculinity, male gender norms, and its association to IPV. FGDs were led by a trained facilitator who guided focus group participants through discussions about community-level norms around masculinity and IPV. We conducted a total of 9 FGDs: 5 FGDs with community members (male; n=49; 2 FGDs with boys, 2 FGDs with young men, and 1 FGD with adult men); and 4 FGDs with key stakeholders who had experience working with local male groups to gather their experiences in working with men and boys implementing IPV prevention programs (male; n=20, female; n=4).

IDIs and FGDs were conducted in the local language of Luganda by professional RHSP interviewers who had prior qualitative research experience. IDI and FGD sessions lasted approximately 1–2 hours. All adult participants, emancipated minors, and the parents of minors provided written informed consent, including consent to audio-record the interview. In Uganda, individuals under the age of 18 were considered emancipated if they were married, had children, or served as the head of a household.^30^ Unemancipated minors under age 18 provided written assent to participate while parents of these minors provided written informed consent. Recorded audio files were transcribed verbatim and translated from Luganda to English. All participants were compensated 10,000 Uganda Shillings (approximately US$3 at the time of data collection) for their time.

### Ethical Clearance

Ethical clearance and approval were provided by Institutional Review Boards at the University of California San Diego Human Research Protections Program and the Uganda Virus Research Institute’s Research Ethics Committee and Uganda National Council for Science and Technology.

### Data Analysis

We used the constructivist grounded theory approach^31^ to develop an analytical framework that reflects how masculinity relates to IPV perpetration in the study setting. We managed transcript analysis using NVivo version 12 Pro (QSR International). In the initial stage of data analysis, 3 coders read transcripts to discover concepts related to masculinity. Thematic analysis was used to develop a coding schema.^32^ To ensure a high correlation between multiple coders, about 10% of data was double-coded. In the second stage of data analysis, axial codes were identified to reflect the relationship among similar concepts identified in the open coding process. We identified 3 axial codes: individual perceptions on masculinity, social norms on masculinity, and context of IPV perpetration. Following identification of axial codes, we selected 2 key conceptual categories on masculinity (i.e., reputation versus respectability) informed by previous qualitative research, constructed out of the data, and based on researchers’ interpretation.^31^

### Analytical Framework: Reputation Versus Respectability

We define masculinity as a set of behaviors that men are socially and culturally encouraged to perform. Collectively, these behaviors are socially, not biologically, constructed and lead to the creation of a gender identity.^33^ It is important to recognize the multiplicity and fluid nature of masculinity and the ways that men and boys relate themselves to collective models of masculinity in a particular setting.^34^ To organize and understand our data, we used the 2 ideals of masculinities, respectability and reputation, that are applied by Siu et al. for their study conducted in a gold mining village in eastern Uganda assessing men’s access to HIV testing.^24^ The 2 constructs of masculinity originated from a study Wilson conducted in the Caribbean.^35^ Respectability ideals include notions of marriage, fathering children and providing for them, sexual fidelity, respect for self and others, and hard work and were generally supported by the wider society, including the majority of women, some men, and religious leaders.^24^ Reputational ideals are endorsed by male peers and include sexual prowess, fathering many children, physical strength, socializing with others, and compulsory spending on leisure.^24^ These 2 key constructs allow us to examine the dynamic interplay of contradicting aspects of masculinities related to VAW (Figure).

**FIGURE:**
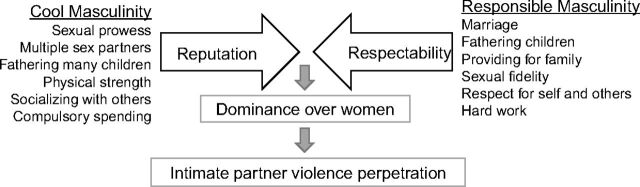
“Reputation” Versus “Respectability” and Intimate Partner Violence Perpetration^a^ ^a^According to Wilson’s (1969) definition, “reputation” is an endorsed perception of male peers and directed toward proficiency in male-centered activities. “Respectability” is conformity to the society and large social institutions such as family and church.^35^ “Cool masculinity” and “responsible masculinity” are emic terms that corresponds to Wilson’s definition and listed characteristics of “cool” and “responsible” masculinities emerged from this qualitative study.

## RESULTS

We described the internal complexity of masculine norms in Rakai by organizing study findings according to 2 masculinity constructs— “respectability” and “reputation”—as shown in the analytical framework (Figure). Both men and boys reported that they were inspired to practice values aligned with attributes related to respectability, but they described how attributes related to reputation were desired among their male peer groups. Further, both masculine norms were associated with the justification of dominance over women and IPV perpetration in situations involving gendered power dynamics (e.g., sexual relationship, sexual fidelity, conflict resolution, and decision making between heterosexual partners).

### Respectability: Responsible Man

Participants across all age groups consistently mentioned that creating a family was the cornerstone of masculinity:

*For a man to be called a man, you must have a family…* —IDI 2, aged 30 years

To assess the ideal image of masculinity, we asked who would be considered a role model in the community. The ideal man was described as someone who has a job and a house, respects and takes care of himself, has a wife and children, and cares for his family. This type of masculine model was often referred to as a “responsible man.” Marriage and reproducing children were viewed as dominant masculine milestones that transitioned a boy into manhood.

*I work for myself. Secondly, I have children, I am a parent and I have work to do which has made me a man.* —IDI 27, aged 20 years

The responsible man was considered to be someone who has a job and a house, respects and takes care of himself, has a wife and children, and cares for his family.

Most participants described that 18 years was a landmark age for males to take on responsibilities, be a man, and have a family.

*This can be considered especially from 18 years of age. He should be able to take care of the family because for anyone to be a man, he should have a family. The young man should own some things [assets], he should be sober or with sound minds and creativity* —IDI 40, aged 49 years

### Reputation: Cool Man

Another recurrent theme was being a “cool man,” or someone who was known for having sexual prowess, multiple sexual partners, and the resources to buy nice things (e.g., fashionable clothing, cars, expensive alcohol for people). Participants explained that “cool men” had money, could afford to buy luxurious items, and were thus entitled to have sex with as many women as desired. As 1 participant described:

*This is someone who buys alcohol for people, he engages in sexual relationship with every girl and actually most people would refer to him as someone with “swagger” [mentions it in English] and for them they think that such a man is so cool.* —IDI 13, aged 34 years

Most participants narrated how they believed that their male peers (not them, personally) perceived having multiple, extramarital, sexual relationships to be masculine. The Luganda phrase, “okutambula-tambula,” which means walking, was brought up repeatedly in reference to married men who have sex with many women. The practice characterized by okutambula-tambula was revered as a masculine and sexually normative behavior for men, but not for women. On the contrary, participants agreed that women who engaged in extramarital affairs were looked down upon in society. Men’s multiple partnering was not only considered manly but also perceived by some to be fully rationalized given the high social value placed on fertility and male virility:

*This is when you have multiple sexual partners and at the time you have children from everywhere you go. This makes you to be considered as a man.* —IDI 8, aged 49 years

The practice characterized by “okutambula-tambula”—which means walking—was revered as a masculine and sexually normative behavior for men, but not for women.

### Context-Specific Dynamics of Masculinities

#### Sexual Relationships

Sexual attraction to women was a social expectation for both men and boys in Rakai. Admiring the physical beauty of women, discussing sex with male peers, initiating sexual relationships with 1 or many women, and having long-lasting sexual experiences were considered hallmarks of masculine sexuality. Participants narrated how, around puberty, a boy should begin discussing and acting on his sexual desire for women, and it was frequently mentioned that a man must initiate sex:

*A man is the one who makes sexual advances when he sees a woman he likes. He can approach that woman and ask her for a talk and then they talk about love issues. After talking about those issues, you have sex. It is the man who starts all that.* —IDI 4, aged 28 years

Participants explained that men who were not engaged in romantic relationships with women would be teased because this behavior was not consistent with prevailing notions of masculinity. They expressed that a lack of sexual prowess was viewed by their peers as sexual weakness, ignorance, or dysfunctionality. Participants used phrases such as “amakaago gaafa” and “yatomerwa endiga” to explain why men cannot have sex with women. Amakaago gaafa means that “your private parts have a health problem, and you cannot have sex with anyone” and yatomerwa endiga translates to “penis death” referring to “someone who lost sexual strength since childhood.” Participants connected masculinity with how long a man could sustain sexual interactions:

*If you ejaculate easily, you are not considered a man…when you ejaculate quickly and the woman stays in need of more sex, time comes and she says that this is not a man.* —IDI 11, aged 45 years

They reported that a majority of their conversations and knowledge about women, sex, and relationships came from their peers or videos on their phones (often referred to as “blue movies”).

*For most young men like us, we learn about romantic relationship through watching “blue movies” (films about pornography). For example, you can get a friend who would start convincing to watch a certain film from which you will learn new techniques about sex.* —FGD 6, Community members aged 18–24 years

Some participants narrated how a “real man” can convince any woman to have sex with him, even if the woman initially denies a sexual relationship:

*She might not have been interested but when you keep luring her, she might end up getting interested. People can even say that this man does not fail. He’s capable of having any woman even those that he fails to get at first, he lies to them until he gets them. Other people will say that she is impossible, how he was able to get her, he is a real man.* —IDI 11, aged 45 years

The value placed on having many children was associated with a negative attitude toward condom use in marriage. Condom use was typically deemed unacceptable unless it was being used to prevent HIV transmission. Since women were expected to be faithful to their husbands, such protection was perceived as only being needed outside of the marriage:

*Sex is natural and it was created to happen and it is what results into having children. But for me I think it would be good for a man to have sex with a condom or after testing for HIV as a way to protect his life. In case he is married and he trusts the partner, they can have sex but remain faithful. So, for those who are not married, it would be good for them to test before having sex or use a condom. Obviously, you cannot tell a married man to use a condom because they must have children.* —IDI 13, aged 34 years

### Violence Perpetration

Men who did endorse exerting dominance over women typically shared that masculine norms related to reputation were encouraged and upheld among their male peers. Concerns related to being ashamed about limited sexual stamina from their female sexual partners were frequently voiced among men. Some justified the use of VAW to recover their dominance and pride as a man:

*Women can undermine you if you cannot full fill their sexual satisfaction for their preferred lengthy period during sex.* —IDI 8, aged 49 years

Men who did endorse exerting dominance over women typically shared that masculine norms related to reputation were encouraged and upheld among their male peers.

Some male participants thought contraception use was linked to women’s loss of sexual desire. Many men perceived hormonal contraceptives as the main reason their wives and sex partners refused to have sex with them, which they felt offended their masculinity. Some men felt justified in using force or violence if one’s wife refused sex:

*There are also women who use family planning methods like Norplant, it reduces a woman’s sexual desire. If you happen to force her into sex, this is likely to cause sexual violence or you may be forced to punish her though it may not have been your intention to cause violence against her.* —IDI 15, aged 45 years

Participants felt that a partner’s covert contraceptive use was emasculating, as they prevented men’s participation in (1) the decision-making process; (2) the ability to produce children; and (3) a feeling that his partner trusts him and is being faithful.

*The family planning methods caused a number of negative side effects among women. For example, there is somebody who may “loss appetite” [loss of sexual desire]. It may result into fighting… in the case your wife did not tell you anything about her decision to use contraceptives. You ask her that please let us play sex, she replies “I am not interested.” What happens next is violence and yet your wife may be so silent to you. Don’t you think that it is a mistake for the woman to fail to tell you [about secret contraceptive use]? It makes you almost feel less of a man*. —IDI 8, aged 49 years

Other factors such as poverty, extramarital sex, or conflicts between a married couple were perceived to threaten men’s respectability in the family, and sometimes led men to justify VAW to regain dominance. For example, IPV was used to assert power when other masculine norms did not work:

*If a man would decide to leave some money at home, then his wife starts complaining that the money is little. He is likely to reply through slapping and kicking a woman as a way of informing her that there is no more money.* —FGD 6, Community members aged 18–24 years

Widespread acceptance and normalization of VAW to control or discipline women were illustrated through most participants’ accounts in the community.

*He can order his wife to do something and eventually she does not do it completely. It is okay when he gives her a slap.* —FGD 1, RHSP counselors

Some men justified IPV within marriage or even considered it necessary when they felt that a wife was not meeting certain expectations placed on her, and they blamed women for provoking such situations. Expectations that men commonly placed on women included obeying her husband and parents-in-law, preparing meals on time, running errands, cleaning the home, and being faithful to her husband.

*After laying down all these rules and the woman fails to adhere, then you are free to give a punishment when she is already aware about it. The right procedure would be the married couple to put into practice what you have agreed upon.* —IDI 35, aged 43 years

Some men justified IPV within marriage or even considered it necessary when they felt that a wife was not meeting certain expectations placed on her.

### Alcohol Use

In general, most participants considered moderate and responsible alcohol use to be a positive masculine ideal. While heavy drinking was considered a hallmark of traditional masculinity, participants described alcohol use beyond one’s control as a promoter of negative behaviors, including engagement in high-risk sex practices and fights. Across all age groups, participants said getting drunk and not being able to control oneself were not manly. Thus, while few participants suggested that alcohol use symbolized masculinity, most participants expressed negative feelings about excessive alcohol use and being drunk.

*A man should not use alcohol because [alcohol] make[s] him unconscious and he engages in violence, or [alcohol] drives him to have sex with bar workers.* —IDI 2, aged 30 years

Alcohol consumption was often described as a trigger for physical and sexual violence, including IPV. Heavy drinking was considered to cause extramarital and transactional sex and conflicts between intimate partners.

*The village men in the communities where we work drink and get intoxicated … He comes back home and knocks the door twice, if they don’t open for him yet he is already “high” like they call it now days, that can make him annoyed and when he quarrels yet he has gotten the wife from her sleep, they will start fighting.* —FGD 4, Health education staff

### Divergence of Masculinity

The divergence of masculinity was observed among younger participants, possibly suggesting a generational shift in masculine norms. Some boys (aged 15–17 years) and young men (aged 18–24 years) had views against having multiple sex partners. The personal beliefs of participants who did not support the prevailing masculinity conflicted with the widespread value placed on men’s prowess and ability to attract and have sex with multiple women.

*A promiscuous man is not a real man. He is a stupid man and always causes problems at his home. He gets involved with many women and picks infections like HIV and infects his wife at home.* —IDI 26, aged 17 years

Some men were able to articulate how masculinity means consensual sex, for an intimate partner or a wife, and not using violence and alcohol to control women.

*For someone to be called a man during sex you should not force your partner or wife to have sex, you should not abuse her, you should not first drink to have sex such that you get into sexual intercourse when you are prepared very well.* —IDI 2, aged 30 years

Because of the extended and strong presence of RHSP HIV prevention programs in Rakai, condom use, circumcision, and HIV testing were often referred to as manly and requisite for healthy relationships.

*It [circumcision] was about protecting myself and in case I get a partner we should use a condom. They added that when I am are going to marry we should first test for HIV and know our status. This is because you can be HIV negative when you are not aware of your partner’s status thus, you must test and get the results together. I don’t engage myself in sexual relationship with different girls. When I think about it and meditate about romantic relationship, I say that God will give me the right person for marriage*. —IDI 13, aged 24 years

Most participants who expressed gender equitable beliefs participated in the 2014 “Stylish Man/Stylish Living” campaign, which aimed to demedicalize HIV prevention messaging and promoted healthy lifestyle choices that included condom use, faithfulness, male circumcision, enrolling or staying in care if HIV positive, treating partners with respect, and caring about health and well-being.^36^ Fourteen of 38 interview participants knew the Stylish Man Campaign that started in Rakai. Our findings imply the effectiveness of the existing gender-transformative program.

## DISCUSSION

This qualitative study used 2 constructs of masculinity, reputation, and respectability to examine the relationship between masculinity and IPV in the rural Rakai region of Uganda. These masculinity constructs were examined in context-specific dynamics in sexual relationships, violence perpetration, and alcohol use. Reputational masculine norms (“cool masculinity”) included becoming sexually active by age 18, being attracted to women, and having high sexual stamina. Respectable masculinity (“responsible masculinity”) was marked by a man’s ability to find a female partner, get married, and have children. We found these 2 constructs of masculinity coexisted in the lives of men and boys depending on the context and reference groups. Specifically, it was important for men and boys to uphold masculine attributes related to their “reputation,” such as having sexual prowess, multiple sexual partners, and resources to buy nice things in male-only peer group settings. However, masculine attributes related to “respectability,” such as having a job, a house, a wife, children, and being able to take care of their family, were highly valued in larger social settings. We found that both masculine constructs could be used to justify dominance over women and perpetration of IPV.

We found that both masculine constructs of reputation and respectability could be used to justify dominance over women and perpetration of IPV.

Under the sexual relationships domain, those who endorsed “reputation” believed in having multiple sex partners, initiating sexual relationships with women with persistence and force, if necessary, and using alcohol. However, those that endorsed “respectability” thought it was more masculine to be monogamous and abstain from alcohol use or drink in moderation. Our findings suggest that a man’s social standing within society is often still measured by the number of sexual partners he has and that this measure is used to evaluate masculinity.

Under the domain of VAW, participants—regardless of what masculinities they endorsed—expressed how violent behavior could be justified to maintain men’s control within their family and sexual relationships. VAW was seen as a way to earn respect from male peers and to avoid being teased. IPV was perceived to be particularly justifiable when a female partner refused to have sex, used contraception covertly, or failed to meet a man’s expectations.

While 2 dominant masculine norms existed in rural Rakai, young men and boys who already participated in gender-transformative programs challenged the existing beliefs, suggesting that some views have already shifted, and thus more can be done to transform these notoriously difficult to change social norms. Some young men and boys aged 15 to 24 years placed a value on gender-equitable behaviors in their relationships, such as open couple communication, nonviolent conflict resolution, and shared decision making. They disagreed with the masculine norms related to reputation and instead held their own masculine beliefs (e.g., sexual fidelity, respect for self, and others). This finding suggests the effectiveness of gender-transformative programs among younger-aged individuals.

Gupta highlighted how power is fundamental to sexuality and gender and introduced gender-transformative approaches to reduce the imbalance in power between women and men.^37^ We delineate 2 masculine norms (reputation and respectability) that endorsed VAW and placed masculinity in a position of power. Our findings corroborate previous research in the region that found that marriage, alcohol, multiple sex partners, sexual entitlement, dominance over women and decision making, and sexual conquest were risk factors for male-perpetrated IPV against women.^12^^,^^38^^–^^40^

### Limitations

This study has limitations that warrant discussion. Participants’ responses could be biased due to social desirability when discussing sexuality and violence perpetration. We tried to mitigate this bias by designing interview and focus group questions that enhanced participants’ comfort to share their personal views without feeling as if any blame or judgment was placed on their individual behaviors. Additionally, while interviews were conducted and transcribed in Luganda, they were translated into English, which could have resulted in mistranslations and loss of cultural nuance, potentially impacting the richness and understanding of participant responses. We addressed this by having all findings reviewed by both members of the research team from Uganda and the United States to increase the trustworthiness of findings by ensuring that the transcripts were interpreted within the cultural context.

## RECOMMENDATIONS

### Gender-Transformative Interventions Should Incorporate Multilevel Approaches to Create Change

We recommend that future gender-transformative interventions incorporate multilevel approaches that target personal, interpersonal, communal, and societal levels of change. Our participants revealed that masculine norms are constructed and reinforced at home, among male peers, and in the larger community, with pressure to adhere to traditional notions of masculinity present across these settings. “Reputation” is emphasized among male peers while “respectability” is valued at home and in the larger community. These norms coexisted in the lives of men depending on the context. These prevailing norms often make men and boys believe in the necessity of violence against a female partner to maintain dominance and respect from one’s wife, peers, and the community at large.

Our participants revealed that masculine norms are constructed and reinforced at home, among male peers, and in the larger community, with pressure to adhere to traditional notions of masculinity present across these settings.

### Safe Spaces Should Be Created to Allow Men to Discuss Challenges

At the personal and interpersonal levels, safe spaces should be formed to allow men to discuss pressure and challenges caused by reputation and respectability.^41^ Gender-transformative intervention activities would benefit from incorporating guidance on using nonviolent conflict-resolution tactics to promote communication and dispel the idea that sexual consent is not masculine. Effective messaging strategies for young adolescent males need to acknowledge the costs of traditional masculinities,^42^^,^^43^ access adults who do not conform to traditional gender roles,^44^ include a family intervention,^45^ and have a gender-equitable male peer group support.^46^^,^^47^ The approach could provide men and boys a chance to reflect on the dynamic, often conflicting, images of men and empower individuals to renegotiate and reconceptualize the masculine norms. Such critical reflection on masculinity could serve as a basis to cultivate qualities related to communication, empathy, and respect that have proven to be effective in other interventions.^42^^,^^46^^–^^50^

### Community-Level Interventions Should Focus on Shifting Norms That Promote Violence

At the community level, gender-transformative programs need to account for the fact that some men use violence to avoid ridicule for diverging from prevailing norms. Since physical and sexual power is an element of masculinity, men may feel the need to assert their power in relationships with women. Community-level interventions should focus on shifting these norms as well as identifying and involving men who do not believe violence is masculine as champions of change.

### Policies Should Be Created and Revised to Shift Societal Norms on Violence

At the societal level, policies should be made and revised to allow for shifts in societal norms that are intolerant of violence.^42^^,^^51^ Resources and manuals such as Manhood 2.0,^52^ A Manual to Spark Critical Reflection on Harmful Gender Norms with Men and Boys in Aquatic Agricultural Systems,^53^ and One Man Can Toolkit^54^ could be adapted to use in school antiviolence programs. Program H developed postcards, banners, comics, and a film promoting a gender-equitable lifestyle among young men and was found to be effective in engaging men and boys in Brazil, India, and Nicaragua.^55^

## CONCLUSION

Our findings provide insights into constructs of masculinities, respectability, and reputation in the context of IPV in rural Uganda. This knowledge can be applied to existing HIV testing, prevention, and care programs, such as the Stylish Man/Stylish Living Campaign^56^ and the Determined, Resilient, Empowered, AIDS-free, Mentored and Safe (DREAMS) partnership^57^ in Rakai. The divergence of masculinity observed from this study implies further research on the effectiveness of existing programs is needed to evaluate how existing gender-transformative programs change masculine norms and IPV in Rakai.

## Notes

**Peer Reviewed**

**First published online:** January 17, 2022.

## References

[1] Reed E Gupta J Silverman JG. Understanding sexual violence perpetration. JAMA Pediatr. 2014;168(6):581–581. 10.1001/jamapediatrics.2013.5408. 24886797 PMC5536096

[2] Jewkes R Fulu E Roselli T Garcia-Moreno C; UN Multi-country Cross-sectional Study on Men and Violence research team. Prevalence of and factors associated with non-partner rape perpetration: findings from the UN Multi-country Cross-sectional Study on Men and Violence in Asia and the Pacific. Lancet Glob Health. 2013;1(4):e208–e218. 10.1016/s2214-109x(13)70069-x. 25104346

[3] Devries KM Mak JY Bacchus LJ. Intimate partner violence and incident depressive symptoms and suicide attempts: a systematic review of longitudinal studies. PLoS Med. 2013;10(5):e1001439. 10.1371/journal.pmed.1001439. 23671407 PMC3646718

[4] Fleming PJ McCleary-Sills J Morton M Levtov R Heilman B Barker G. Risk factors for men’s lifetime perpetration of physical violence against intimate partners: results from the international men and gender equality survey (IMAGES) in eight countries. PLoS One. 2015;10(3):e0118639. 10.1371/journal.pone.0118639. 25734544 PMC4348538

[5] Stöckl H Devries K Rotstein A. The global prevalence of intimate partner homicide: a systematic review. Lancet. 2013;382(9895):859–865. 10.1016/S0140-6736(13)61030-2. 23791474

[6] Campbell JC. Health consequences of intimate partner violence. Lancet. 2002;359(9314):1331–1336. 10.1016/S0140-6736(02)08336-8. 11965295

[7] The Global Health Observatory. World Health Organization; 2021. Updated May 6, 2021. Accessed December 13, 2021. https://www.who.int/data/gho/data/indicators/indicator-details/GHO/intimate-partner-violence-lifetime

[8] Ellsberg M Arango DJ Morton M. Prevention of violence against women and girls: what does the evidence say? Lancet. 2015;385(9977):1555–1566. 10.1016/S0140-6736(14)61703-7. 25467575

[9] Barker F Ricardo C Nascimento M. *Engaging Men and Boys in Changing Gender-Based Inequity in Health: Evidence From Programme Interventions*. World Health Organization; 2007. Accessed December 6, 2021. https://www.who.int/gender/documents/Engaging_men_boys.pdf

[10] World Health Organization (WHO). *Violence Against Women Prevalence Estimates, 2018: Global, Regional and National Prevalence Estimates for Intimate Partner Violence Against Women and Global and Regional Prevalence Estimates for Non-partner Sexual Violence Against Women*. WHO; 2021. Accessed December 6, 2021. https://www.who.int/publications/i/item/9789240022256

[11] Casey E Carlson J Two Bulls S Yager A. gender transformative approaches to engaging men in gender-based violence prevention: a review and conceptual model. Trauma Violence Abuse. 2018;19(2):231–246. 10.1177/1524838016650191. 27197533

[12] Fleming PJ Lee JGL Dworkin SL. “Real men don’t”: constructions of masculinity and inadvertent harm in public health interventions. Am J Public Health. 2014;104(6):1029–1035. 10.2105/AJPH.2013.301820. 24825202 PMC4062033

[13] Dworkin SL Treves-Kagan S Lippman SA. Gender-transformative interventions to reduce HIV risks and violence with heterosexually-active men: a review of the global evidence. AIDS Behav. 2013;17(9):2845–2863. 10.1007/s10461-013-0565-2. 23934267

[14] Kyegombe N Abramsky T Devries KM. The impact of SASA! a community mobilization intervention, on reported HIV-related risk behaviours and relationship dynamics in Kampala, Uganda. J Int AIDS Soc. 2014;17(1):19232. 10.7448/IAS.17.1.19232. 25377588 PMC4223282

[15] Goldmann L Lundgren R Welbourn A. On the CUSP: the politics and prospects of scaling social norms change programming. Sexual and Reproductive Health Matters. 2019;27(2):51–63. 10.1080/26410397.2019.1599654. 31533586 PMC7887930

[16] Jewkes R Nduna M Levin J. Impact of Stepping Stones on incidence of HIV and HSV-2 and sexual behaviour in rural South Africa: cluster randomised controlled trial. BMJ. 2008;337:a506. 10.1136/bmj.a506. 18687720 PMC2505093

[17] Sharma V Leight J Verani F Tewolde S Deyessa N. Effectiveness of a culturally appropriate intervention to prevent intimate partner violence and HIV transmission among men, women, and couples in rural Ethiopia: findings from a cluster-randomized controlled trial. PLoS Med. 2020;17(8):e1003274. 10.1371/journal.pmed.1003274. 32810146 PMC7433859

[18] Vaillant J Koussoubé E Roth D Pierotti R Hossain M Falb KL. Engaging men to transform inequitable gender attitudes and prevent intimate partner violence: a cluster randomised controlled trial in North and South Kivu, Democratic Republic of Congo. BMJ Glob Health. 2020;5(5):e002223. 10.1136/bmjgh-2019-002223. 32467354 PMC7259847

[19] Wagman JA King EJ Namatovu F. Combined intimate partner violence and HIV/AIDS prevention in rural Uganda: design of the SHARE intervention strategy. Health Care Women Int. 2016;37(3):364–387. 10.1080/07399332.2015.1061526. 26086189 PMC5039238

[20] Wagman JA Gray RH Campbell JC. Effectiveness of an integrated intimate partner violence and HIV prevention intervention in Rakai, Uganda: analysis of an intervention in an existing cluster randomised cohort. Lancet Glob Health. 2015;3(1):e23–e33. 10.1016/S2214-109X(14)70344-4. 25539966 PMC4370228

[21] Wagman JA Namatovu F Nalugoda F. A public health approach to intimate partner violence prevention in Uganda: the SHARE Project. Violence Against Women. 2012;18(12):1390–1412. 10.1177/1077801212474874. 23419276

[22] Wagman JA Gray RH Nakyanjo N. Process evaluation of the SHARE intervention for preventing intimate partner violence and HIV infection in Rakai, Uganda. Eval Program Plann. 2018;67:129–137. 10.1016/j.evalprogplan.2017.12.009. 29310019 PMC6821387

[23] Nyanzi S Nyanzi-Wakholi B Kalina B. Male promiscuity. Men Masc. 2009;12(1):73–89. 10.1177/1097184X07309503

[24] Siu GE Wight D Seeley JA. Masculinity, social context and HIV testing: an ethnographic study of men in Busia district, rural eastern Uganda. BMC Public Health. 2014;14(1):33. 10.1186/1471-2458-14-33. 24417763 PMC3893584

[25] Siu GE Seeley J Wight D. Dividuality, masculine respectability and reputation: How masculinity affects men’s uptake of HIV treatment in rural eastern Uganda. Soc Sci Med. 2013;89:45–52. 10.1016/j.socscimed.2013.04.025. 23726215

[26] Siu GE Wight D Seeley J. ‘Dented’ and ‘resuscitated’ masculinities: the impact of HIV diagnosis and/or enrolment on antiretroviral treatment on masculine identities in rural eastern Uganda. Sahara J. 2014;11(1):211–221. 10.1080/17290376.2014.986516. 25444303 PMC4272191

[27] Wawer MJ Gray RH Sewankambo NK. A randomized, community trial of intensive sexually transmitted disease control for AIDS prevention, Rakai, Uganda. AIDS. 1998;12(10):1211–1225. 10.1097/00002030-199810000-00014. 9677171

[28] Wawer MJ Sewankambo NK Serwadda D. Control of sexually transmitted diseases for AIDS prevention in Uganda: a randomised community trial. Rakai Project Study Group. Lancet. 1999;353(9152):525–535. 10.1016/S0140-6736(98)06439-3. 10028980

[29] Chang LW Grabowski MK Ssekubugu R. Heterogeneity of the HIV epidemic in agrarian, trading, and fishing communities in Rakai, Uganda: an observational epidemiological study. Lancet HIV. 2016;3(8):e388–e396. 10.1016/S2352-3018(16)30034-0. 27470029 PMC4973864

[30] Uganda National Council for Science and Technology (UNCST). *National Guidelines for Research Involving Humans as Research Participants*. UNCST; 2014. Accessed December 6, 2021. https://iuea.ac.ug/sitepad-data/uploads//2021/03/Human-Subjects-Protection-Guidelines-July-2014.pdf

[31] Charmaz K. Constructionism and the grounded theory method. In: Holstein JA Gubrium JF, eds. *Handbook of Constructionist Research*. The Guilford Press; 2008:397–412. Accessed December 6, 2021. http://www.sxf.uevora.pt/wp-content/uploads/2013/03/Charmaz_2008-a.pdf

[32] Tuckett AG. Applying thematic analysis theory to practice: a researcher’s experience. Contemp Nurse. 2005;19(1-2):75–87. 10.5172/conu.19.1-2.75. 16167437

[33] Courtenay WH. Constructions of masculinity and their influence on men’s well-being: a theory of gender and health. Soc Sci Med. 2000;50(10):1385–1401. 10.1016/S0277-9536(99)00390-1. 10741575

[34] Connell R. Margin becoming centre: for a world-centred rethinking of masculinities. NORMA. 2014;9(4):217–231. 10.1080/18902138.2014.934078

[35] Wilson PJ. Reputation and respectability: a suggestion for Caribbean ethnology. Man. 1969;4(1):70–84. 10.2307/2799265

[36] Kankaka EN Ssekasanvu J Prodger J. Sexual risk behaviors following circumcision among HIV-positive men in Rakai, Uganda. AIDS Care. 2018;30(8):990–996. 10.1080/09540121.2018.1437253. 29433386 PMC6284241

[37] Gupta GR. Gender, sexuality, and HIV/AIDS: the what, the why, and the how. Can HIV AIDS Policy Law Rev. 2000;5(4):86–93. 11833180

[38] Abramsky T Devries K Kiss L. A community mobilisation intervention to prevent violence against women and reduce HIV/AIDS risk in Kampala, Uganda (the SASA! Study): study protocol for a cluster randomised controlled trial. Trials. 2012;13(1):96. 10.1186/1745-6215-13-96. 22747846 PMC3503643

[39] Mullinax M Grilo SA Song XS. HIV-risk behaviors of men who perpetrate intimate partner violence in Rakai, Uganda. AIDS Educ Prev. 2017;29(6):527–539. 10.1521/aeap.2017.29.6.527. 29283273 PMC6710836

[40] Kouyoumdjian FG Calzavara LM Bondy SJ. Risk factors for intimate partner violence in women in the Rakai Community Cohort Study, Uganda, from 2000 to 2009. BMC Public Health. 2013;13(1):566–566. 10.1186/1471-2458-13-566. 23759123 PMC3701515

[41] Pulerwitz J Barker G Segundo M Nascimento M. *Promoting More Gender-Equitable Norms and Behaviors Among Young Men as an HIV/AIDS Prevention Strategy*. Population Council; 2006. Accessed December 6, 2021. https://promundoglobal.org/resources/promoting-more-gender-equitable-norms-and-behaviors-among-young-men-as-an-hivaids-prevention-strategy/

[42] Viitanen AP Colvin CJ. Lessons learned: program messaging in gender-transformative work with men and boys in South Africa. Glob Health Action. 2015;8(1):27860. 10.3402/gha.v8.27860. 26350433 PMC4563102

[43] Barker G. Gender equitable boys in a gender inequitable world: reflections from qualitative research and program development with young men in Rio de Janeiro, Brazil. Sex Relationship Ther. 2000;15(3):263–282. 10.1080/14681990050109854

[44] Pulerwitz J Barker G. Measuring attitudes toward gender norms among young men in Brazil. Men Masc. 2008;10(3):322–338. 10.1177/1097184X06298778

[45] Dworkin SL Hatcher AM Colvin C Peacock D. Impact of a gender-transformative HIV and antiviolence program on gender ideologies and masculinities in two rural, South African communities. Men Masc. 2013;16(2):181–202. 10.1177/1097184X12469878. 24311940 PMC3848879

[46] Peacock D Barker G. Working with men and boys to prevent gender-based violence. Men Masc. 2014;17(5):578–599. 10.1177/1097184X14558240

[47] van den Berg W Hendricks L Hatcher A Peacock D Godana P Dworkin S. ‘One Man Can’: shifts in fatherhood beliefs and parenting practices following a gender-transformative programme in Eastern Cape, South Africa. Gend Dev. 2013;21(1):111–125. 10.1080/13552074.2013.769775. 24000276 PMC3758761

[48] MacPhail C Khoza N Treves-Kagan S. Process elements contributing to community mobilization for HIV risk reduction and gender equality in rural South Africa. PLoS One. 2019;14(12):e0225694. 10.1371/journal.pone.0225694. 31790483 PMC6886772

[49] Stern E Pascoe L Shand T Richmond S. Lessons learned from engaging men in sexual and reproductive health as clients, partners and advocates of change in the Hoima district of Uganda. Cult Health Sex. 2015;17(sup2):190–205. 10.1080/13691058.2015.1027878. 25953243 PMC4706030

[50] Ratele K. Working through resistance in engaging boys and men towards gender equality and progressive masculinities. Cult Health Sex. 2015;17(sup2):144–158. 10.1080/13691058.2015.1048527. 26073936 PMC4706035

[51] Fleming PJ Gruskin S Rojo F Dworkin SL. Men’s violence against women and men are inter-related: recommendations for simultaneous intervention. Soc Sci Med. 2015;146:249–256. 10.1016/j.socscimed.2015.10.021. 26482359 PMC4643362

[52] Abebe KZ Jones KA Culyba AJ. Engendering healthy masculinities to prevent sexual violence: rationale for and design of the Manhood 2.0 trial. Contemp Clin Trials. 2018;71:18–32. 10.1016/j.cct.2018.05.017. 29802967 PMC6643273

[53] Promundo-US; CGIAR Research Program on Aquatic Agricultural Systems. *Promoting Gender-Transformative Change With Men and Boys: A Manual to Spark Critical Reflection on Harmful Gender Norms With Men and Boys in Aquatic Agricultural Systems*. Promundo-US and CGIAR Research Program on Aquatic Agricultural Systems; 2016. Accessed December 6, 2021. https://promundoglobal.org/resources/promoting-gender-transformative-change-men-boys/

[54] One Man Can toolkit. Sonke Gender Justice Network. Accessed December 6, 2021. https://genderjustice.org.za/project/community-education-mobilisation/one-man-can/one-man-can-toolkit/

[55] Flood M. Involving men in efforts to end violence against women. Men Masc. 2011;14(3):358–377. 10.1177/1097184X10363995

[56] *Promising Practice Uganda: Rakai “Stylish Man” Campaign: Combining Traditional and New Approaches to Demand Creation for Safe Male Circumcision (SMC)*. Clearinghouse on Male Circumcision for HIV Prevention. Rakai Health Sciences Programme; 2014. https://www.malecircumcision.org/file/61332/download?token=AeHh0GIV

[57] Matovu JKB Nambuusi A Nakabirye S Wanyenze RK Serwadda D. Formative research to inform the development of a peer-led HIV self-testing intervention to improve HIV testing uptake and linkage to HIV care among adolescents, young people and adult men in Kasensero fishing community, Rakai, Uganda:a qualitative study. BMC Public Health. 2020;20(1):1582–1582. 10.1186/s12889-020-09714-1. 33081735 PMC7576713

